# FOXM1 contributes to treatment failure in acute myeloid leukemia

**DOI:** 10.1172/jci.insight.121583

**Published:** 2018-08-09

**Authors:** Irum Khan, Marianna Halasi, Anand Patel, Rachael Schultz, Nandini Kalakota, Yi-Hua Chen, Nathan Aardsma, Li Liu, John D. Crispino, Nadim Mahmud, Olga Frankfurt, Andrei L. Gartel

**Affiliations:** 1 Department of Medicine, University of Illinois, Chicago, Illinois, USA.; 2 Department of Medicine and; 3 Department of Pathology, Northwestern University, Chicago, Illinois, USA.; 4 Department of Pathology, University of Illinois, Chicago, Illinois, USA.; 5 Division of Epidemiology and Biostatistics, School of Public Health, University of Illinois, Chicago, Illinois, USA.

**Keywords:** Hematology, Oncology, Cancer, Drug therapy

## Abstract

Acute myeloid leukemia (AML) patients with NPM1 mutations demonstrate a superior response to standard chemotherapy treatment. Our previous work has shown that these favorable outcomes are linked to the cytoplasmic relocalization and inactivation of FOXM1 driven by mutated NPM1. Here, we went on to confirm the important role of FOXM1 in increased chemoresistance in AML. A multiinstitution retrospective study was conducted to link FOXM1 expression to clinical outcomes in AML. We establish nuclear FOXM1 as an independent clinical predictor of chemotherapeutic resistance in intermediate-risk AML in a multivariate analysis incorporating standard clinicopathologic risk factors. Using colony assays, we show a dramatic decrease in colony size and numbers in AML cell lines with knockdown of FOXM1, suggesting an important role for FOXM1 in the clonogenic activity of AML cells. In order to further prove a potential role for FOXM1 in AML chemoresistance, we induced an FLT3-ITD–driven myeloid neoplasm in a FOXM1-overexpressing transgenic mouse model and demonstrated significantly higher residual disease after standard chemotherapy. This suggests that constitutive overexpression of FOXM1 in this model induces chemoresistance. Finally, we performed proof-of-principle experiments using a currently approved proteasome inhibitor, ixazomib, to target FOXM1 and demonstrated a therapeutic response in AML patient samples and animal models of AML that correlates with the suppression of FOXM1 and its transcriptional targets. Addition of low doses of ixazomib increases sensitization of AML cells to chemotherapy backbone drugs cytarabine and the hypomethylator 5-azacitidine. Our results underscore the importance of FOXM1 in AML progression and treatment, and they suggest that targeting it may have therapeutic benefit in combination with standard AML therapies.

## Abstract 2

Nuclear FOXM1 confers resistance to standard acute myeloid leukemia chemotherapy and may represent a potential therapeutic target.

## Introduction

The prevalent backbone for the initial treatment of AML is a 2-drug chemotherapy regimen comprising an anthracycline and cytarabine with response rates of 40%–70%. Over half of patients relapse within 3 years. With improved treatment-related mortality, emergence of resistance to chemotherapeutic drugs represents a key issue in the treatment of AML. There is an unmet need for new pathogenic models of resistance that could provide targets for therapeutic intervention.

The nuclear-cytoplasmic shuttle, nucleophosmin (NPM1), is mutated in one-third of AML cases, leading to its aberrant cytoplasmic sequestration (1). Our work focuses on harnessing the favorable clinical outcomes seen in NPM1 mutant AML (2, 3) in response to conventional chemotherapy. We have shown that, across multiple tumor types, NPM binds to FOXM1, and their interaction (4) is required for sustaining the level and localization of FOXM1.

FOXM1 is an oncogenic transcription factor belonging to the Forkhead family of transcription factors (5). FOXM1 regulates a variety of processes critical to cancer progression, including tumorigenesis, cell proliferation (6, 7), metastasis, angiogenesis, and chemoresistance (8). The FOXM1 network was shown to be the foremost predictor of adverse outcomes in a PRECOG dataset of 18,000 cancers (9). FOXM1 mediates resistance to genotoxic agents, such as γ-irradiation and epirubicin, through regulating genes (10, 11) involved in DNA damage repair. Targeting FOXM1 sensitizes breast (12), gastric, and ovarian (13) cancer cells to chemotherapy, as well as lung cancer (14) and B cell leukemia (15) to tyrosine kinase inhibition.

FOXM1 is regulated through protein expression, posttranslational modification, and subcellular localization. We have linked the mechanism underlying the chemosensitivity conferred by the NPM1 mutation in AML to the cytoplasmic relocalization and consequent inactivation of FOXM1. Our previous work suggests that nuclear FOXM1 expression is diminished in the favorable-risk NPM1 mutant AML, and its inhibition in NPM1 WT cells results in sensitization to conventional chemotherapy (16).

In this study, we examine the clinical relevance of FOXM1 as a predictor of chemoresistance in intermediate-risk AML, a group comprising >50% of newly diagnosed AML cases. The therapeutic approach to these patients at this time is relatively uniform in spite of significant heterogeneity in outcomes. Genomic profiling has attempted to fragment this category to allow more risk-adapted treatment approaches (17). However interpreting the prognostic effects of individual mutations in AML is impeded by their interactions with other driver mutations (18).

Moreover, there is a dearth of targetable mediators of chemotherapy resistance. While we use cytogenetics and a handful of well-validated somatic mutations to recognize a high risk of relapse, we are lagging in a targeted therapeutic approach to these patients. Attempts at targeting NPM have been thwarted by its promiscuous molecular interactions (19), resulting in pro- and antioncogenic roles (20, 21). By focusing on its binding partner FOXM1 with selective overexpression in transformed cells and oncogenic properties, we introduce a potentially novel target-directed approach to sensitize AML cells to chemotherapy. This therapeutic approach could be applicable to all AML patients with WT NPM1.

## Results

### FOXM1 nuclear overexpression can predict failure of induction chemotherapy in AML.

Patients >18 years of age with AML with intermediate-risk cytogenetics (22) were enrolled in this multiinstitution retrospective study. We focused on intermediate-risk patients where there is an unmet need for discerning prognostic markers. Clinical characteristics are summarized in Table 1. There were a total of 111 patients with a median age of 61 years, with an interquartile range of 51–69 years. There was no sex bias, and the combined population of the 2 institutions had broad racial and ethnic representation, with 51% patients of nonhispanic white, 18% black, 12% hispanic, and 19% subjects reported as other. The median BM blast percentage of 60% reflected that the biopsies were heavily infiltrated with leukemia so the imaging data was reflective of the cells of interest. A total of 87% of the patients had a normal karyotype. Consistent with published prevalence data, 42 of the 100 patients (42%) who underwent molecular testing had an NPM1 mutation, and 31 of 107 patients tested (28%) had a FLT3-ITD mutation (23).

Treatment details are shown in Table 1. Induction therapy included cytarabine and an anthracycline in 88 cases and hypomethylating agents (HMA) in 16 cases. Five patients were managed with palliative care, and 2 were transferred to other institutions. Twenty-nine subjects were enrolled on clinical trials for induction therapy. These included SWOG-1203, ECOG-2906, AAML1031, CALGB 10603, and investigator-initiated trials.

**Figure 1:**
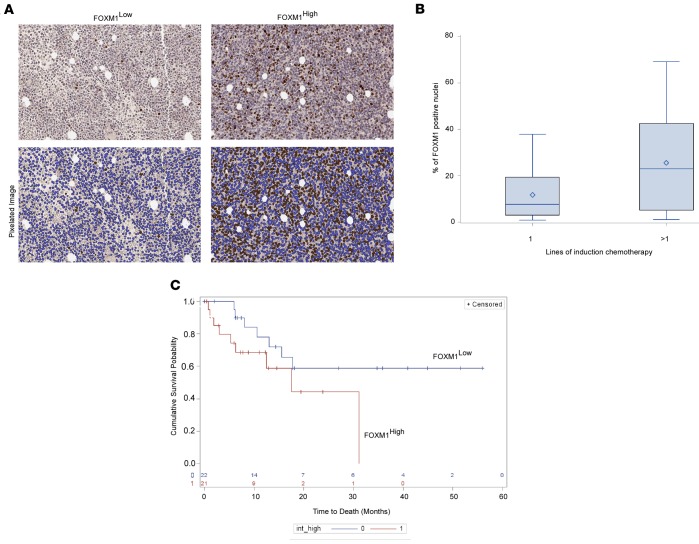
FOXM1 is an independent predictor of chemotherapy resistance in intermediate risk CN-AML. (**A**) BM slides were stained with FOXM1 antibody and counterstained with hematoxylin. Utilizing the Aperio AT2 whole slide scanner and HALO 2.0 software, images were analyzed. Two representative patient samples are shown (200× magnification) with high and low percentage of nuclei expressing FOXM1, with the corresponding markup images below that were quantified. (**B**) In an analysis of the patients who achieved a CR following chemotherapy, there were 74 BM samples with quantifiable FOXM1 expression. We found that patients needing more than 1 cycle of induction therapy had greater than a 2-fold increase in the percentage of nuclei expressing FOXM1 (mean 25.6% vs. 11.4% nuclei, *P* = 0.004, 2-tailed *t* test) in their diagnostic BM. (**C**) Kaplan-Meier analysis for overall survival in 43 patients from a single institution in our cohort, stratified based on average nuclear intensity of FOXM1. FOXM1^hi^ patients had an inferior survival that approached statistical significance (median 501 days vs. not reached, *P* = 0.068, log rank test).

Out of 88 patients treated with chemotherapy, 80 (91%) eventually achieved a complete remission with or without count recovery (CR or CRi, respectively) as defined by International Working Group criteria (24). Lines of induction therapy needed to achieve remission served as a measure of chemotherapy resistance. Patients were stratified as needing 1 or >1 line of induction therapy. FOXM1 expression data was available on the biopsies of 74 patients who achieved a remission, and representative images are shown from low- and high-expressing subjects (Figure 1A) with the corresponding pixilated image that is quantified.

There were 50 patients of the 74 who achieved a CR with 1 cycle of induction chemotherapy and 24 patients who required >1 cycle. We found that patients needing >1 line of induction therapy had more than a 2-fold increase in the percentage of nuclei expressing FOXM1 in their BM biopsy compared with responding patients (mean 25.6% vs. 11.4% nuclei, *P* = 0.004) (Figure 1B). The average nuclear intensity of FOXM1 was also significantly higher in patients who failed their first line of induction (mean OD, 0.22 vs. 0.16; *P* = 0.02). In regression analysis, the percentage of FOXM1-positive nuclei significantly predicted resistance to first-line chemotherapy with an odds ratio (OR) of 1.80 for a 10% increase in positive nuclei (*P* = 0.005). The average nuclear intensity of FOXM1 in the pretreatment BM was also a significant predictor of chemotherapy resistance (OR 2.5 for 0.1 U increase in OD, *P* = 0.02). In the multivariate logistic regression model (Table 2) assessing the effects of FOXM1 variables on resistance to first-line chemotherapy, we adjusted clinical-pathologic risk factors including age, WBC count at presentation, and presence of the FLT3-ITD mutation. Due to interinstitution variability in consolidation strategies, survival analysis was done independently for each institution. FOXM1 nuclear/cytoplasmic (N:C) ratio, as well as average nuclear FOXM1 intensity, were able to predict inferior overall survival (OS) in a single institution cohort (*n* = 43) (Figure 1C) using Cox regression analysis (HR = 4.7 for every 0.1 unit increase in N:C ratio, *P* = 0.03; HR = 4.27 for every 0.1 unit increase in OD, *P* = 0.06). In addition, in this single-institution survival analysis, FOXM1 N:C ratio was an independent predictor of OS in a multivariate analysis including FLT3-ITD, NPM1 mutation, BMI, age, and WBC (Supplemental Figure 1; supplemental material available online with this article; https://doi.org/10.1172/jci.insight.121583DS1).

In summary, within the cytogenetically homogenous group of intermediate-risk AML patients, FOXM1 nuclear expression as a quantitative variable is able to distinguish a population at risk of treatment resistance and possible inferior survival.

### Transgenic overexpression of FOXM1 confers chemotherapy resistance in myeloid neoplasms.

**Figure 2:**
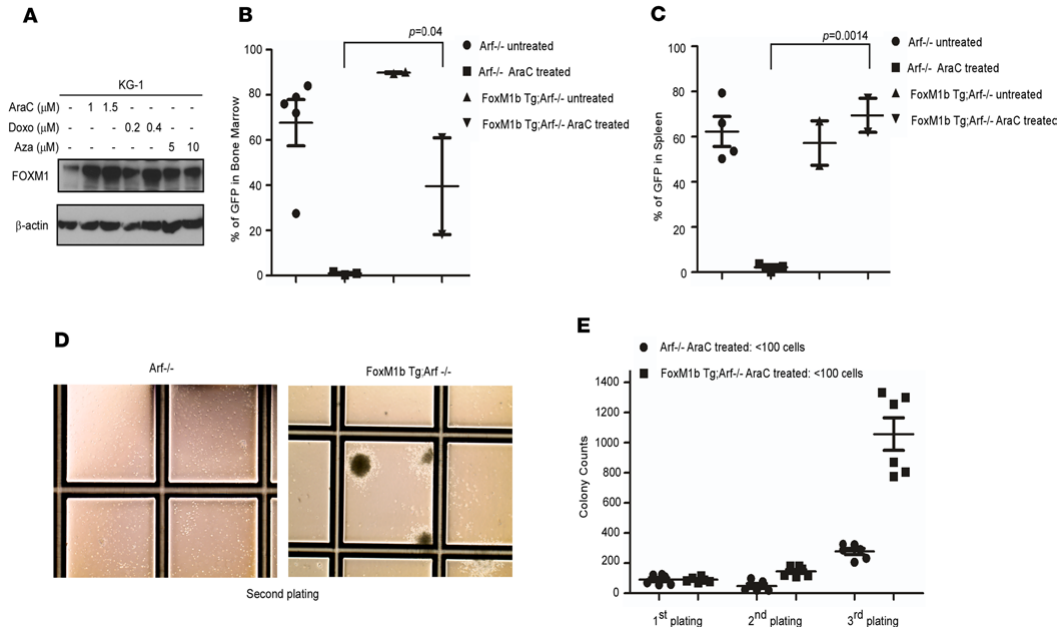
FOXM1 confers resistance to standard chemotherapy. (**A**) KG-1 cells were treated as indicated. Total cell lysates were analyzed by Western blotting for the level of FOXM1. (**B** and **C**) Transgenic FOXM1 overexpressing (FoxM1b Tg;Arf^–/–^) and control (Arf^–/–^) animals were treated with 5-FU to enrich for hematopoietic progenitor cells. These cells were transduced with FLT3-ITD retroviral particles and transplanted into syngeneic recipients. Following disease establishment, animals were randomized and treated with vehicle or cytarabine (AraC) for 5 consecutive days. Three weeks after treatment, the BM (**B**) and the spleens (**C**) were analyzed for leukemic burden as assessed by GFP measurement by flow cytometry. Data are expressed as the mean ± SEM (*n* = 4/group); *P* < 0.05 by unpaired one-tailed *t* test (**B**) and unpaired 2-tailed *t* test (**C**). (**D**) FLT3-ITD transformed BM cells (generated and treated as described in **B** and **C**) were studied in serial replating colony assays. Representative images of the colonies were imaged with the EVOS XL Core Imaging System using the 4× objective. (**E**) Plot shows increased colony numbers with the serial replating in the treated FOXM1 overexpressing mice compared with their treated control counterparts. Data are expressed as the mean ± SEM (*n* = 2) experiments done in triplicate; *P* < 0.05 by unpaired 2-tailed *t* test.

The effect of standard-of-care AML therapies on the expression of FOXM1 was investigated. Using a panel of AML cell lines including KG-1 (Figure 2A), HL-60, and THP-1 (Supplemental Figure 2, A and B), we show clear evidence of FOXM1 upregulation in the total cell lysate within 24 hours of exposure to chemotherapy. This rapid upregulation of FOXM1 in response to most standard therapies used in the treatment of AML suggests that this may be a common mechanism of resistance utilized by AML cells.

To establish whether FOXM1 regulates chemotherapy resistance in myeloid leukemia, we utilized previously developed transgenic mice lacking the alternative reading frame (ARF) tumor suppressor: (a) Arf^–/–^ C57BL/6 and (b) Rosa26-FoxM1b Tg;Arf^–/–^ C57BL/6 (25). We are utilizing FOXM1 expression in the Arf^–/–^ background to overcome sustained expression of ARF, which is a potent inhibitor of FOXM1 transcriptional activity. Following 5-fluorouracil (5-FU) treatment, hematopoietic progenitor cells (HPCs) from both Arf^–/–^ and FoxM1b Tg;Arf^–/–^ mice were collected and transduced with FLT3-ITD retrovirus and transplanted into lethally irradiated recipients. All the animals developed a myeloproliferative neoplasm (MPN) characterized by leukocytosis and splenomegaly, as previously described (26). Engraftment was similar between genotypes, as confirmed by peripheral blood (PB) chimerism on day 14, and animals were randomized to treatment with vehicle versus cytarabine 75 mg/kg i.p. for 5 consecutive days. The animals were sacrificed 3 weeks following treatment, and blood, BM, and spleen were analyzed for disease assessment. The MPN developed on a FOXM1-overexpressing background showed significantly higher resistance to standard chemotherapy, as evidenced by increased leukemic burden in the BM, spleen, and blood by GFP assessment (Figure 2, B and C, and Supplemental Figure 2C) and by larger spleen size (Supplemental Figure 2D). Peripheral smears and blood counts show persistently elevated circulating myeloid cells (Supplemental Figure 2, E and F) following treatment in the FOXM1-overexpressing model.

Given the putative role of the leukemia-initiating cells (LICs) in mediating relapse, we also explored the effect of chemotherapy on the LIC population in the context of FOXM1 overexpression. In a parallel experiment, BM mononuclear cells (MNCs) from FOXM1-overexpressing and control FLT3-ITD leukemic mice were plated in colony assays 1 week following cytarabine treatment. To assess the residual burden of LICs in the FoxM1b Tg;Arf^–/–^ mice compared with Arf^–/–^ littermates, we did serial replating assays. Serial replating resulted in increasing plating efficiency in the cells overexpressing FOXM1 that may contribute to the chemotherapeutic resistance in these animals (Figure 2, D and E). The rapid resurgence of disease and increased replating ability within weeks of chemotherapy treatment suggests FOXM1 is a critical mediator in the emergence of resistant leukemic clones.

### Ixazomib is a potentially novel FOXM1 inhibitor.

**Figure 3:**
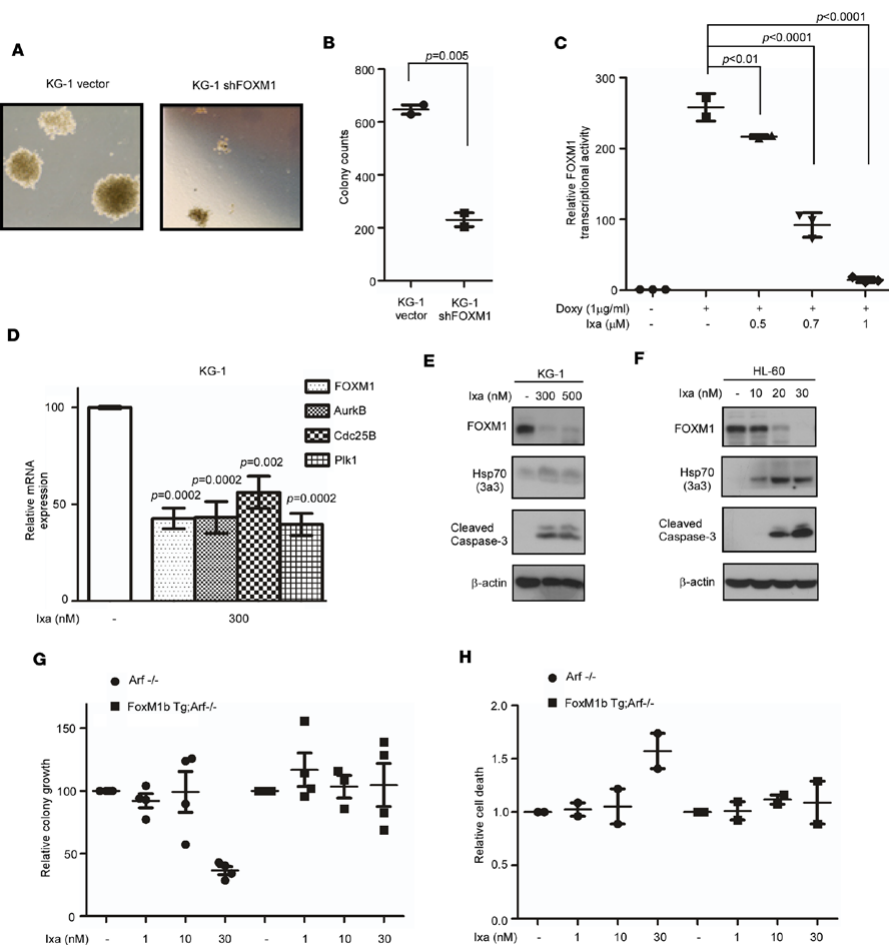
Ixazomib inhibits FOXM1 activity. (**A**) KG-1 FOXM1-knockdown cells show smaller colony size and reduced colony numbers compared with cells transduced with empty vector in a methylcellulose assay. Colonies were imaged with the EVOS XL Core Imaging System using the 4× objective. (**B**) Plot represents the mean ± SEM of 3 independent colony assay experiments, each plated in duplicate. *P* < 0.05 by unpaired 2-tailed *t* test. (**C**) Ixazomib inhibits FOXM1 transcriptional activity in an inducible luciferase cell line. The luciferase activity was determined by using the Luciferase Assay System (Promega). We show significant dose-dependent inhibition of FOXM1 transcriptional activity. Plot shows quantification as fold induction of firefly luciferase activity compared with control cells, mean ±SD of a representative triplicate experiment. *P* < 0.05 by 1-way ANOVA followed by Tukey’s multiple comparison post test. (**D**) Ixazomib-treated and untreated KG-1 leukemia cells were collected for RNA extraction. Quantitative PCR was carried out with FOXM1, AurkB, Cdc25B, and Plk1 primers. Graph shows quantification as percentage of mRNA expression levels in treated cells compared with control cells; mean ± SEM of 3 independent experiments. *P* < 0.05 by 1-way ANOVA followed by Tukey’s multiple comparison post test. (**E** and **F**) In a panel of AML cell lines (KG-1 and HL-60), FOXM1 protein expression was suppressed by treatment with ixazomib, as detected by immunoblotting. This also correlated with stabilization of HSP-70 and caspase-3 cleavage. (**G** and **H**) FLT3-ITD–transformed primary murine BM cells (generated as described in Figure 2, B and C) were sorted and treated as indicated and studied in colony and apoptotic assays, as assessed by flow cytometry after annexin V–PE staining. Plots show resistance to ixazomib treatment in FOXM1-overexpressing BM cells compared with their treated control counterparts. Data are expressed as the mean ± SEM (*n* = 2/group); *P* < 0.05 by 1-way ANOVA followed by Tukey’s multiple comparison post test.

Having established the role of FOXM1 in mediating chemotherapy resistance in myeloid neoplasms, we went on to inhibit this transcription factor. First, leukemia cell lines with stable knockdown of FOXM1 were generated as previously published (27). Using methycellulose-based colony assays, we noted a dramatic decrease in colony size and numbers in KG-1 (Figure 3, A and B) and MV-4-11 cell lines with knockdown of FOXM1 (Supplemental Figure 3, A and B), suggesting an important role for FOXM1 in the colony-forming activity of AML cells.

We have previously shown that FOXM1 is a general target of proteasome inhibitors (PIs) (28). The mechanism underlying this effect is stabilization of HSP70, which is a negative regulator of FOXM1 (29). Ixazomib is an oral PI that is approved for the treatment of relapsed multiple myeloma and is very well tolerated (30). Second, the PI ixazomib was tested for in vitro effect on FOXM1 in AML cells. Ixazomib inhibits the transcriptional activity of FOXM1 using a luciferase reporter osteosarcoma cell line with inducible FOXM1 (Figure 3C). We then assessed the mRNA levels of FOXM1 and its transcriptional targets. Treatment with ixazomib resulted in dose-dependent inhibition of FOXM1 and its transcriptional targets aurora kinase B (AurkB), Cdc25B, and polo-like kinase 1 (Plk1) (Figure 3D and Supplemental Figure 3C). FOXM1 binds to its own responsive regulatory elements as part of an autoregulation loop (31), and therefore, FOXM1 mRNA levels are also downregulated following ixazomib treatment. Dose-dependent FOXM1 protein inhibition by ixazomib is accompanied by stabilization of HSP-70 and increasing levels of apoptosis in KG-1, HL-60 (Figure 3, E and F), and SET-2 (Supplemental Figure 3D) cell lines. Our data suggest that, in AML, ixazomib at nanomolar doses is a potent FOXM1 inhibitor, which may be contributing to its antileukemic activity. We did additional experiments using BM MNCs from FLT3-ITD–induced leukemia in FOXM1-overexpressing and control transgenic mice. The GFP-positive transformed cells were sorted and subsequently exposed to a range of doses of ixazomib from 1–30 nM and plated in colony-forming assays (Figure 3G), and they were examined for cell death at 24 hours by flow cytometry (Figure 3H). The results show that overexpression of FOXM1 reverses the antileukemic activity of ixazomib, thereby implicating FOXM1 in mediating the antileukemic activity of this drug.

### Ixazomib inhibits FOXM1 in primary AML samples and induces apoptosis.

**Figure 4:**
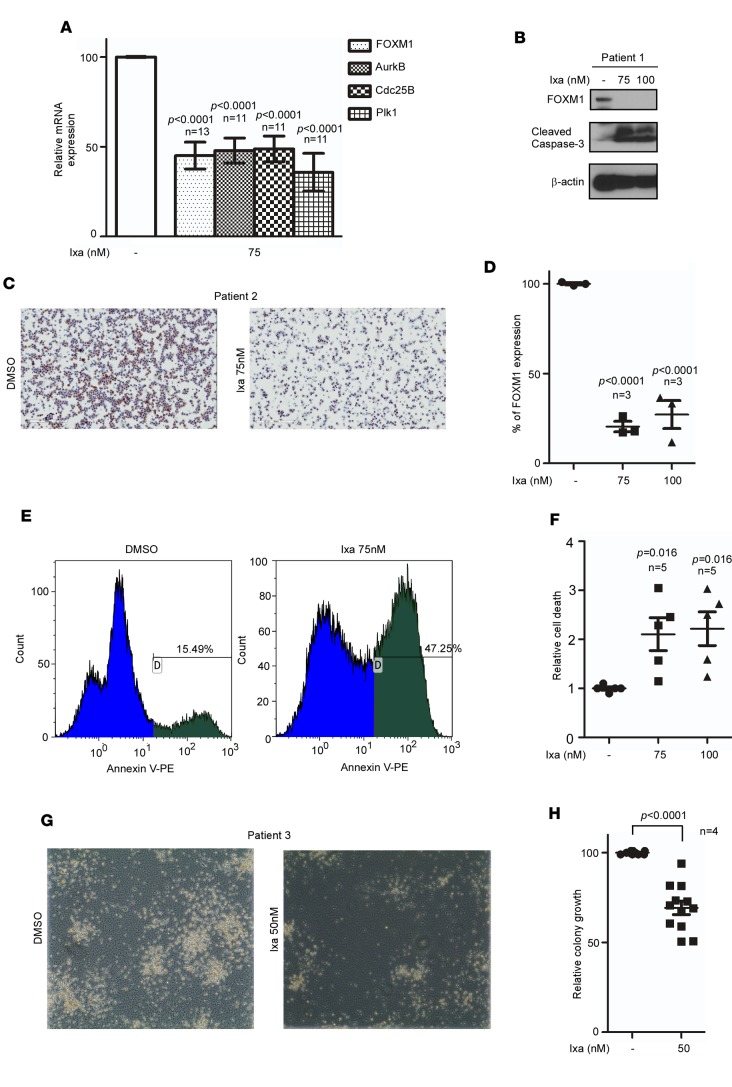
Ixazomib inhibits FOXM1 and induces cell death in primary AML cells. (**A**) AML mononuclear cells show significant downregulation of FOXM1 mRNA expression, as well as its downstream targets (AurkB, Cdc25B, and Plk1) following 24-hour treatment in liquid culture, as assessed by quantitative PCR. Data are expressed as the mean ± SEM (*n* = 11–13 patients); *P* < 0.05 by 1-way ANOVA followed by Tukey’s multiple comparison post test. (**B**) A representative Western blot from a newly diagnosed patient with complex karyotype AML (80% circulating blasts) shows significant FOXM1 protein inhibition after ixazomib treatment ex vivo with induction of cell death by caspase-3 cleavage. (**C**) Cytospins prepared from the mononuclear cells isolated from patients with relapsed AML show high nuclear expression of FOXM1 in blast cells and downregulation after 24-hour incubation with ixazomib (100× magnification). (**D**) Plot shows quantification of nuclear FOXM1 in AML cytospin slides by the Aperio microscope as a percentage compared with DMSO-treated cells; mean ± SEM of 3 different patient samples. *P* < 0.0001 by 1-way ANOVA followed by Tukey’s multiple comparison post test. (**E**) Treatment with 75 nM of ixazomib for 24 hours is associated with markedly increased apoptosis, as shown by a representative histogram of annexin V–PE staining. (**F**) Plot shows fold increase in cell death by flow cytometry after annexin V–PE staining compared with control cells; mean ± SEM of 5 patient samples . *P* < 0.05 by 1-way ANOVA followed by Tukey’s multiple comparison post test. (**G**) CFU assay confirms inhibition of colony-forming activity in peripheral blood mononuclear cells from AML patients following treatment with ixazomib. Colonies were imaged with the EVOS XL Core Imaging System using the 4× objective. (**H**) Plot shows quantification as the percentage of colony growth compared with control cells ± SEM of 4 independent triplicate experiments; *P* < 0.0001 by unpaired 2-tailed *t* test.

MNCs were used in ex vivo studies to evaluate the effects of ixazomib on inhibition of FOXM1 in patient-derived AML cells. We assessed 13 patient samples treated in liquid culture. Cells were incubated with DMSO or 75 nM ixazomib for 24 hours and then RNA extracted. The mean of 13 samples is shown, with FOXM1 mRNA expression normalized to DMSO-treated cells. Ixazomib causes significant downregulation of FOXM1 mRNA (Figure 4A), as well as FOXM1 canonical targets AurkB, Cdc25B, and Plk1 in primary AML cells. Inhibition of FOXM1 protein expression was confirmed by Western blot with associated induction of apoptosis, as detected by caspase 3 cleavage (Figure 4B). Cytospin preparations of AML MNCs treated ex vivo with ixazomib show significant downregulation of nuclear FOXM1 by IHC (Figure 4, C and D).

Treatment of patient samples (*n* = 5) with ixazomib resulted in a 2-fold increase in cell death by annexin staining (Figure 4, E and F). Colony assays show inhibition of colony-forming activity in 4 patient samples (Figure 4, G and H). These findings in primary AML cells recapitulate the observation in cell lines that ixazomib inhibits FOXM1, resulting in inhibition of colony-forming activity and induction of apoptosis.

### Ixazomib has antitumor activity in vivo in a s.c. xenograft model of AML.

The anticancer activity of ixazomib was evaluated in AML xenografts. Six-week-old male athymic nude mice were injected s.c. with HL-60 cancer cells in the flank region bilaterally to establish xenograft tumors. After tumors became palpable, their size was measured by calipers and the animals were randomized to vehicle (cyclodextran) or ixazomib (8 mg/kg) treatment. Mean tumor volume was equivalent between groups. Animals were administered the drug 2 times per week by oral gavage. Tumor size was recorded twice a week. After sacrifice, their tumors were excised.

**Figure 5:**
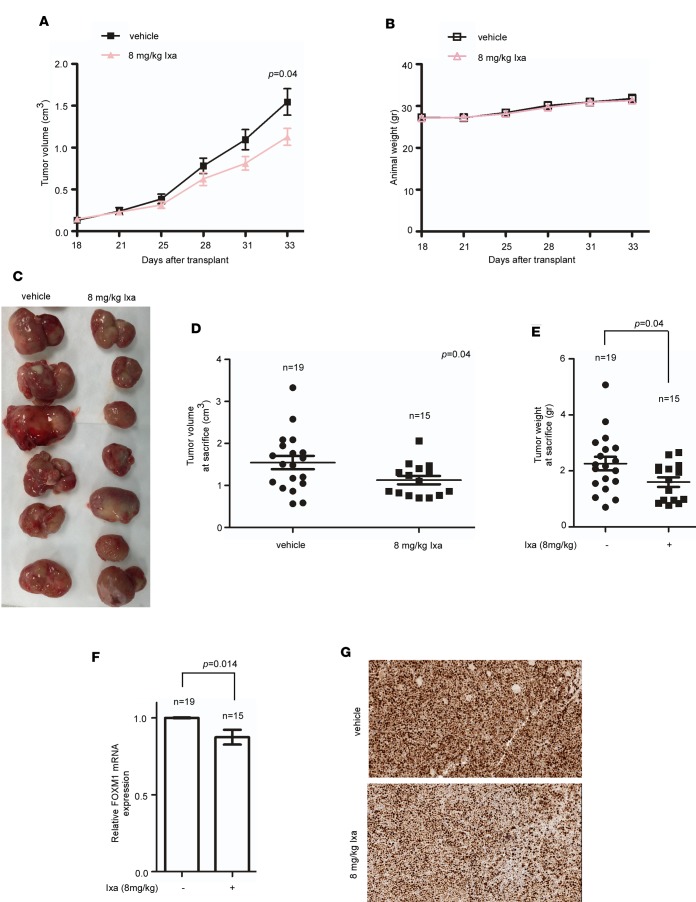
Ixazomib inhibits tumor growth in a s.c. xenograft model of AML. (**A**) HL-60 cells were implanted into the flanks of nude mice bilaterally (*n* = 10/group). Treatment with vehicle versus ixazomib 8 mg/kg by oral gavage 2 times a week was commenced 2 weeks following implantation. After 3 weeks of treatment, the animals were sacrificed. The animals treated with ixazomib displayed slowed tumor growth over serial time points. *P* < 0.05 by unpaired 2-tailed *t* test. (**B**) Graph shows the change in weight of the animals during the treatment period. (**C**) Representative image of the excised tumors is shown. (**D**) Ixazomib-treated animals showed decreased tumor volume at the time of sacrifice. Data are expressed as the mean ± SEM (*n* = 15–19 tumors/group); *P* < 0.05 by unpaired 2-tailed *t* test. (**E**) Treated animals also had significantly decreased tumor weight at sacrifice. *P* < 0.05 by unpaired 2-tailed *t* test. (**F**) FOXM1 mRNA expression was downregulated in ixazomib-treated animals. *P* < 0.05 by unpaired 2-tailed *t* test. (**G**) Also, FOXM1 protein levels decreased following treatment with ixazomib, as detected by IHC (200× magnification).

The mean tumor volume was plotted against time to compare growth kinetics over 3 weeks (Figure 5A). We also found that the dose used for this study did not induce any undue toxic effects, and animals in both groups had similar body weights at the time of sacrifice (Figure 5B). Ixazomib-treated animals showed decreased tumor volume at the time of sacrifice (Figure 5, C and D), as well as reduced tumor mass (Figure 5E). To demonstrate on-target effect of ixazomib, FOXM1 mRNA expression (Figure 5F), and FOXM1 protein by IHC (Figure 5G) were evaluated and showed downregulation. These results support the in vitro studies with ixazomib and strengthen the contention that ixazomib administered by the oral route has antitumor activity in AML that is associated with FOXM1 suppression.

### Ixazomib ameliorates leukemia burden in an orthotopic model of AML, and this correlated with FOXM1 suppression.

We next determined whether ixazomib could decrease leukemic burden and improve outcomes in an orthotopic murine model of AML. KG-1 cells were injected into the tail veins of sublethally radiated immunodeficient (NOD-SCID-γ–null; NSG) mice. At day 14, the PB was analyzed for human CD45 expression (huCD45) by flow cytometry, and any animal with >2% engraftment was included. Animals were randomized into control and treatment groups, and the treatment group received ixazomib (8 mg/kg) i.v. twice a week for 4 weeks. At the end of treatment, the entire cohort was sacrificed, and BM and PB involvement by AML cells was quantified by flow cytometry for huCD45 expression.

**Figure 6:**
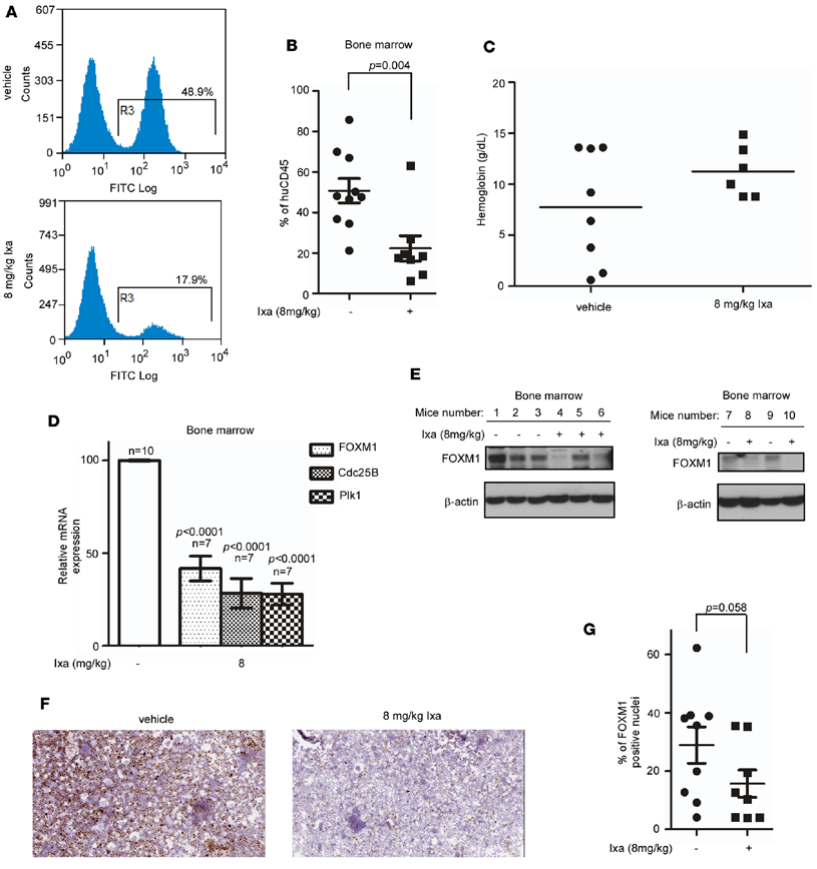
Ixazomib shows antileukemic activity and inhibits FOXM1 in an orthotopic AML model with improved hematopoiesis. (**A**) NSG mice were treated with 8 mg/kg ixazomib i.v. twice a week for 4 weeks. Ixazomib treatment resulted in substantial reduction of leukemic disease burden in the BM as assessed by CD45 expression. Representative flow plots are shown from each group. (**B**) Plot shows quantification of CD45 expression in vehicle- and ixazomib-treated animals. Data are expressed as the mean ± SEM (*n* = 8–10/group); *P* < 0.05 by unpaired 2-tailed *t* test. (**C**) Peripheral blood was analyzed to study the effects on normal blood production. Treated animals showed a higher hemoglobin count, suggesting improved hematopoiesis. (**D**) Following treatment, BM cells were collected for RNA extraction. FOXM1, Cdc25B, and Plk1 mRNA expression levels using quantitative PCR were quantified as percentage of mRNA expression levels in treated cells compared with control cells. Data are expressed as the mean ± SEM (*n* = 7/group); *P* < 0.05 by 1-way ANOVA followed by Tukey’s multiple comparison post test. (**E**) FOXM1 inhibition in the BM cells is shown by Western blotting. (**F**) Cytospins from the BM mononuclear cells were prepared, and representative images are shown (200× magnification). (**G**) Nuclear FOXM1 was quantified in ixazomib- and vehicle-treated animals. Animals treated with ixazomib had significant downregulation of nuclear FOXM1. Data are expressed as the mean ± SEM (*n* = 8/group); *P* = 0.058 by unpaired 1-tailed *t* test.

Ixazomib reduced the leukemia burden in the BM of treated animals (Figure 6, A and B). We also did note an increase in mean hemoglobin from 7.75 g/dl to 11.25 g/dl (Figure 6C) in the treated animals, suggesting improved hematopoiesis. The dot plot shows the hemoglobin range in the vehicle-treated animals with several animals with hemoglobin < 5g/dl. In comparison, none of the drug-treated animals had such profound anemia. The mean platelet count was similar between groups (data not shown), suggesting ixazomib did not suppress normal hematopoiesis. The antileukemic effect of ixazomib with relative sparing of normal hematopoiesis, as evidenced by the improved anemia levels, makes this drug a promising agent in the treatment of AML. To further establish that ixazomib does not suppress normal hematopoiesis, healthy 6-week-old NSG mice were randomized and treated with 8 mg/kg ixazomib or vehicle i.v. twice a week for 4 weeks. The treated animals showed no perturbations in weight or hematological parameters in the PB compared with their vehicle-treated counterparts. This suggests that doses of ixazomib that suppress FOXM1 and attenuate leukemia disease severity do not disrupt normal hematopoiesis (Supplemental Figure 4).

Ixazomib treatment over 4 weeks also significantly inhibited FOXM1 and its transcriptional targets in BM MNCs (Figure 6D). This was corroborated by suppression of FOXM1 protein in the BM MNCs, as shown by Western blot (Figure 6E). Cytospins were also prepared from MNCs and showed marked inhibition of nuclear FOXM1 expression in the treated animals (Figure 6F). Quantitative analysis of nuclear FOXM1 expression in the BM cytospin slides showed a marked reduction in the mean percentage of nuclei expressing FOXM1 from 29% in vehicle-treated animals (*n* = 9) to 16% in ixazomib treated animals (*n* = 8; *P* = 0.058 using a 1-tailed *t* test; Figure 6G). By utilizing the minimally effective dose of the drug needed to target FOXM1, it has the potential to be safely combined with other chemotherapeutic agents without significant myelotoxicity.

### Ixazomib sensitizes AML cells to standard chemotherapeutics.

**Figure 7:**
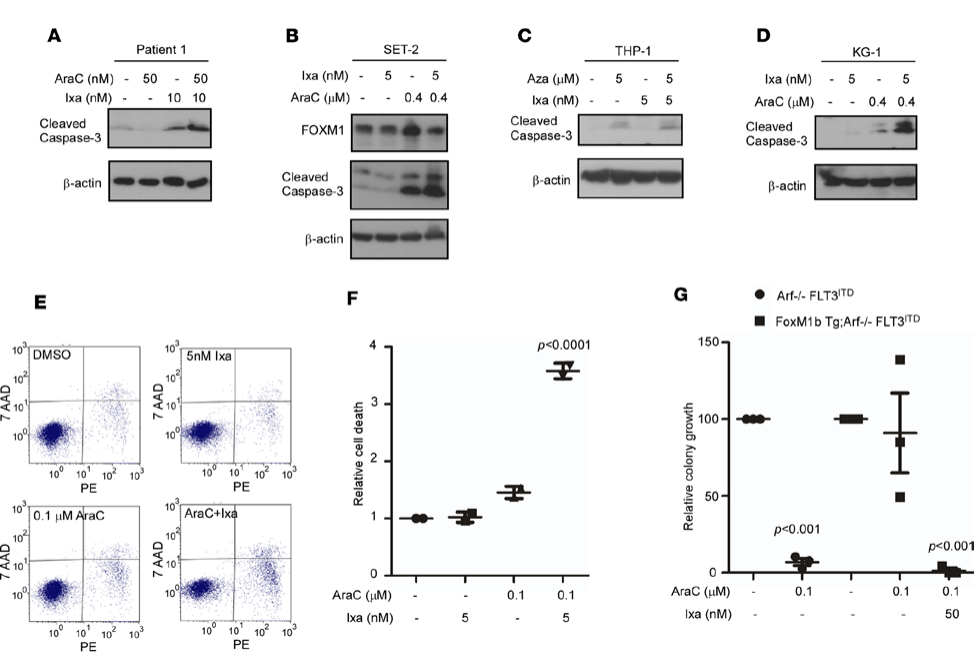
Ixazomib sensitizes leukemia cells to low doses of cytotoxic chemotherapy drugs. (**A–C**) Complex karyotype AML MNCs, as well as SET-2 and THP-1 cells, were treated for 24 hours with the indicated concentrations of AraC or Aza singly and in combination with ixazomib. Combination treatment resulted in increased cell death. (**D** and **E**) KG-1 and MV-4-11 cells were preincubated with ixazomib overnight. Then, ixazomib was removed and cells were treated with Arac for 24 hours. Cell death was elevated after combination treatment, as assessed by caspase-3 cleavage and by annexin V–PE staining. (**F**) Dot plot shows fold increase in cell death by flow cytometry after annexin V–PE staining compared with control cells; mean ± SD of a representative duplicate experiment. *P* < 0.0001 by 1-way ANOVA followed by Tukey’s multiple comparison post test. (**G**) FLT3-ITD–transformed BM cells (generated as described in Figure 2, B and C) were treated as indicated and studied in colony assays. Dot plot shows sensitization to standard chemotherapy with the combination of ixazomib treatment in FOXM1-overexpressing BM cells. Data are expressed as the mean ± SEM of a triplicate experiment; *P* = 0.001 by 1-way ANOVA followed by Tukey’s multiple comparison post test.

Lastly, we studied the effects of low-dose ixazomib to increase sensitivity of leukemia cells to standard chemotherapy drugs. Having shown that most commonly utilized chemotherapeutic agents induce expression of FOXM1 in AML cells (Figure 2A and Supplemental Figure 2, A and B), we used low doses of ixazomib to suppress FOXM1 and enhance the antileukemic activity of these drugs. Using AML MNCs and SET-2 and THP-1 cell lines, we show synergistic induction of apoptosis with low-dose cytarabine or 5-azacitidine in combination with ixazomib (Figure 7, A–C). We also investigated pretreatment of AML cells KG-1 and MV-4-11 with ixazomib for 18 hours and confirmed that this brief exposure to a FOXM1 inhibitor was sufficient to induce sensitization to subsequent treatment with cytarabine, as assessed by caspase-3 cleavage (Figure 7D) and by annexin staining (Figure 7, E and F).

We have also shown chemosensitization using FLT3-ITD leukemia developed in a transgenic FOXM1-overexpressing model (Figure 7G). We sorted FLT3-ITD-GFP–transduced HPCs in an Arf^–/–^ or FoxM1b Tg; Arf^–/–^background. The sorted cells were grown in the presence of cytarabine in colony-forming assays. The FOXM1-overexpressing cells were resistant to the dose of 100 nM cytarabine. Ixazomib was added to the FOXM1-overexpressing leukemic cells and conferred susceptibility to the low-dose cytarabine. Thus, we show that the addition of low-dose ixazomib overcomes the resistance conferred by FOXM1 overexpression.

## Discussion

The application of targeted therapies in specific molecular subsets of AML patients is allowing a survival benefit to emerge, resulting in several new drug approvals in the past 2 years (17). However, the setback in relying purely on genomic classification to allocate patients to therapeutic pathways is the complexity of AML genomes, the observation that most patients harbor multiple gene mutations, and the dynamic patterns of disease evolution (18). Instead of targeting specific genetic aberrations, an alternate strategy for treatment would be to target more commonly dysregulated pathways that are implicated across AML subtypes.

The recent success of a liposomal particle with fixed-ratio delivery of cytarabine and daunorubicin (Vyxeos) shows that utilizing innovative approaches, currently approved agents can be dramatically enhanced in their efficacy. Recognizing a potential mechanism of resistance in the patient at diagnosis would help tailor the treatment regimen to be more effective and increase remission rates. Current genomic predictors of resistance to induction chemotherapy in AML include mutations in RUNX1, ASXL1, and TP53; elevated SNP-A–based genomic complexity; and specific recurrent copy number aberrations/loss of heterozygosity (32).

Our work draws attention to a potentially novel predictor of chemotherapeutic resistance in AML. FOXM1 is a transcription factor expressed in proliferating cells, but not in quiescent or terminally differentiated cells, making it an attractive target. The relevance of FOXM1 in AML is supported by an important bedside to bench discovery, where we previously showed that the favorable NPM1 mutant subset of AML had reduced nuclear levels of FOXM1 (16). The current work establishes the prognostic relevance of nuclear FOXM1 in AML in a clinical retrospective analysis of over 100 patients. Previous publications have observed increased FOXM1 in high-risk molecular subsets of AML (33), but it has not been validated as a prognostic marker in a clinical cohort until now.

Using quantitative microscopy on the diagnostic BM biopsies of a multiinstitution cohort, we confirmed the clinical significance of the nuclear expression of this protein in predicting outcomes. When nuclear FOXM1 expression was combined in a multivariate analysis with conventional clinical and molecular predictors of outcome, it emerged as an independent predictor of upfront chemotherapeutic resistance. Moreover, when the institutions were considered individually, nuclear FOXM1 expression emerged as a predictor of inferior OS (Figure 1).

Using a transgenic murine model of FOXM1 overexpression, we provide proof of concept that FOXM1 overexpression induces resistance to the AML chemotherapy backbone cytarabine (Figure 2). Transcription factors have conventionally been considered as undruggable targets. Our study has challenged this dogma and provides evidence that targeting FOXM1 has antileukemic effects (Figures 5 and 6). We used 2 methods to decrease FOXM1 activity in AML: RNA interference, which is specific, and PIs, which are clinically approved but relatively nonspecific (Figure 3 and Supplemental Figure 2). PIs act by stabilizing HSP70, which we have shown to be a negative regulator of FOXM1 (29).

Here, we establish that ixazomib, an oral PI, has antileukemic activity that correlates with the inhibition of FOXM1 (Figures 5 and 6). Decreasing FOXM1 activity in human and murine leukemia cells with WT NPM1 led to decreased clonogenicity and increased apoptosis (Figures 3 and 4). Additionally, treatment with ixazomib was very well tolerated and reduced tumor burden in vivo in several AML models (Figures 5 and 6). Recent work has demonstrated a critical role of FOXM1 in maintaining hematopoietic stem cell quiescence (34). We postulate that there may be differential levels of FOXM1 expression in leukemic stem cells compared with hematopoietic stem cells that would allow for a therapeutic window to target FOXM1. This is the subject of ongoing work.

PIs have already entered the clinical realm of AML. Previous trials have demonstrated therapeutic benefit by adding bortezomib to cytarabine-based chemotherapy (35–37), but they increased toxicity. In addition to being the first oral PI, ixazomib is well tolerated with minimal neurotoxicity and myelosuppression (38). We show that low-dose ixazomib induces sensitization of AML cell lines and primary human and murine AML cells to the chemotherapy backbone drugs cytarabine and 5-azacitidine (Figure 7).

The putative mechanism of action of PIs is the inhibitory effect on transcription factor NF-κB through stabilization of its inhibitor, I-κB (39). We present an alternate mechanism of action of ixazomib and link its antileukemic activity to its effects on FOXM1. Using overexpression of FOXM1, we show the antileukemic activity of ixazomib is, at least in part, through its inhibition of FOXM1.

In summary, our study provides proof of principle for the inhibition of the transcription factor FOXM1 as a potentially novel strategy in the treatment of AML. Currently approved therapies such as the PI ixazomib could be harnessed to overcome FOXM1-mediated resistance in AML. Moreover, our findings demonstrate the need for the development of more specific and potent FOXM1 inhibitors in the treatment of leukemia.

## Methods

### Patients.

Adult patients diagnosed with intermediate-risk AML were identified at Northwestern Memorial and the University of Illinois Hospitals between 2003 and 2017 using pathology databases (Sunquest). A total of 111 patients were included in the study. Clinical data was collected, and corresponding BM biopsy samples were retrieved under an IRB approved protocol and reviewed by a hematopathologist to ensure adequacy of samples.

### Imaging analysis.

FOXM1-stained slides were scanned on a Aperio AT2 whole slide scanner (Leica Biosystems) at 20× magnification. The images were analyzed using HALO 2.0 software (Indica Labs). The regions of interest (ROI) containing hematopoietic cells were manually selected, and any tissue or staining artifacts were excluded from analysis by manual drawing. Hematoxylin counterstain was used to segment nuclei within the ROIs and to establish an accurate cell count. Threshold values were set for each slide to determine nuclei positive for the FOXM1 marker. FOXM1 staining intensity in the cytoplasm of each cell was measured within a radius of approximately 1 μm grown around each nucleus. The nuclear and cytoplasmic FOXM1 intensity, as well as their ratio, was calculated per each cell and averaged per each slide.

### BM sample processing and IHC.

Tissue sections were stained on Bond RX autostainer (Leica Biosystems) following a preset protocol. In brief, sections were deparaffinized, subjected to EDTA-based (Bond ER2 solution, pH 9) antigen retrieval for 40 minutes at 100°C and blocked with hydrogen peroxide for 5 minutes. After washing with Bond Wash Solution, sections were incubated with anti-FOXM1 antibody (1:250, Abcam, ab207298) for 30 minutes. The detection was performed using Bond Polymer Refine Detection kit (Leica Biosystems, DS9800). All slides were counterstained with Mayer’s hematoxylin for 5 minutes and mounted with Surgipath Micromount Media (Leica Biosystems).

### Cell Culture.

KG-1 (ATCC), HL-60 (ATCC), and MV-4-11 (ATCC) human cell lines were grown in IMDM medium (Thermo Fisher Scientific); THP-1 (ATCC) and SET-2 (ATCC) human cells were grown in RPMI (Thermo Fisher Scientific); and C3-luc cell line (22) was grown in DMEM (Thermo Fisher Scientific). Media were supplemented with 10% FBS (Atlanta Biologicals) and 1% penicillin-streptomycin (Thermo Fisher Scientific). Stable FOXM1-knockdown cell lines using the ATCC obtained parental cells were generated as described in ref. 23. All cells were maintained at 37°C in 5% CO_2_. Cytarabine (MilliporeSigma) and ixazomib (Takeda) were dissolved in DMSO for cell culture experiments; Azacitidine (Wockhardt) and doxycycline (LKT Laboratories) were dissolved in PBS. By company recommendation, ixazomib was dissolved in 5% cyclodextran (MilliporeSigma) for the in vivo animal experiments.

### Immunoblot analysis.

Treated cells were harvested and lysed using IP buffer (20 mM HEPES, 1% Triton X-100, 150 mM NaCl, 1 mM EDTA, 1 mM EGTA, 100 mM NaF, 10 mM Na_4_P_2_O_7_, 1 mM sodium orthovanadate, 0.2 mM PMSF supplemented with protease inhibitor tablet; Roche Applied Sciences). Protein concentration was determined by the Bio-Rad Protein Assay reagent (Bio-Rad). Isolated proteins were separated on SDS-PAGE and transferred to PVDF membrane (MilliporeSigma). Immunoblotting was carried out with antibodies specific for FOXM1 (Santa Cruz Biotechnology Inc.; Novus), cleaved caspase-3 (Cell Signaling Technology), and β-actin (MilliporeSigma). The respective image blots are from the same parts of the same gel and at the same exposures.

### Primary cells.

After Ficoll centrifugation, MNCs were treated in culture at a density of 0.5 × 10^6^ /ml in StemSpan with CD34 expansion supplement (Stemcell Technologies) for 24 hours. Cells were then harvested for RNA extraction, Western blot, cytospin preparation, or analysis of apoptosis by flow cytometry.

### Flow cytometry.

To quantify the level of leukemic engraftment in the orthotopic AML xenograft model, we labeled the PB and BM MNCs with the anti–huCD45-FITC (BD Pharmingen, catalog 555482) antibody following red cell lysis. The cells, which had been previously washed with PBS, were stained for 30 minutes at 20°C. After washing, the samples were analyzed with the Gallios flow cytometer (Becton Dickinson).

Similarly for the FLT3-ITD–transduced primary murine HPCs, the level of engraftment in the BM and spleens of primary recipients was analyzed based on GFP expression.

For apoptosis analysis, harvested cells were stained using the annexin V–PE apoptosis detection kit (BD Pharmingen). Cells were washed with PBS and then resuspended in 100 μl of binding buffer and incubated with PE-conjugated annexin V and 7AAD. The mixture was incubated at room temperature for 15 minutes before flow cytometric analysis with the Cyan ADP instrument (Beckman Coulter).

### Colony-forming cell assay.

AML cell lines KG-1 and MV-4-11 stably transfected with control or FOXM1 shRNA were utilized for colony-forming assays done in triplicate. All experiments were duplicated. Methoccult H4230 (Stem Cell Technologies) was used.

For primary AML samples, MNCs isolated by Ficoll density centrifugation were utilized, and 2 × 10^4^ cells were plated in each condition in Methoccult H4434 (Stem Cell Technologies). At days 12–14, colonies were enumerated. Colony imaging was performed using EVOS cell imaging system (Thermo Fisher Scientific).

For replating assays, we used murine FLT3-ITD BM cells harvested 1 week following cytarabine treatment. We plated 1 × 10^4^ MNCs per plate in Methoccult M3434 and counted at days 5–7 after each plating. Colony imaging was performed using the EVOS XL Core cell imaging system.

### Luciferase assay.

Cells were treated as indicated in the figure legends. The luciferase activity was determined by the Luciferase Assay System (Promega) according to the recommendations of the manufacturer.

### Total RNA extraction and quantitative PCR.

To extract total RNA, cells were collected by TRIzol reagent (Invitrogen). Complementary DNA (cDNA) was synthesized using the High Capacity cDNA Reverse Transcription Kit (Applied Biosystems). Quantitative PCR (qPCR) was run using the ABI 7900 HT (Applied Biosystems) machine with primers, as follows: FOXM1-sense (S), 5′-GGA GGA AAT GCC ACA CTT AGC G-3′, FOXM1-antisense (AS), 5′-TAG GAC TTC TTG GGT CTT GGG GTG-3′; AurkB-S, 5′-ATC TGC TCT TAG GGC CAA GGG-3′, AurkB-AS, 5′-CAC ATT GTC TTC CTC CTC AGG G-3′; Cdc25B-S, 5′-TCC TCC GCT CAA AAT CAC TGT G-3′, Cdc25B-AS, 5′-TGC TGA ACT TGC CCG TCA ATA G-3′; Plk1-S, 5′-ACG GCT TTT TCG AGG ACA AC-3′, Plk1-AS, TGG CAG CCA AGC ACA ATT TG-3′; and 18S rRNA-S, 5′-CGA AGA CGA TCA GAT ACC GT-3′, 18S rRNA-AS, 5′-GGT CAT GGG AAT AAC GCC G-3′.

### IHC on cytospins.

Cytospins were prepared per standard protocol. Slides were stained on Bond RX autostainer (Leica Biosystems) following a preset protocol. Cells were prefixed with ethanol for 10 minutes, followed by blocking with hydrogen peroxide for 15 minutes and Background Sniper blocking reagent (Biocare Medical) for 15 minutes. After washing with Bond Wash Solution, samples were incubated with anti-FOXM1 antibody (1:250, Abcam, ab207298) for 30 minutes. The detection was performed using Bond Polymer Refine Detection kit (Leica Biosystems, DS9800). All slides were counterstained with Mayer’s hematoxylin for 5 minutes and mounted with Surgipath Micromount Media (Leica Biosystems).

### S.c. xenografts.

Four-week-old, male, athymic, nude mice were purchased from Taconic. Bilaterally, 2 × 10^6^ HL-60 cells per site in a 100-μl mixture of 70% Matrigel (BD Biosciences) and 30% PBS were injected s.c. in the flank region of mice. After tumors became palpable, tumor size was measured twice a week using a vernier caliper, and tumor volume was calculated with the following formula: (length × width × height)/2. When tumors reached 70 mm^3^ in size, mice were divided into 2 groups: control (5% cyclodextran) and ixazomib (8 mg/kg), administered by oral gavage. Ixazomib was dissolved in 5% cyclodextran. All drugs were administered by oral gavage 2 times per week for 3 weeks. At the completion of the study, mice were sacrificed by CO_2_ inhalation, followed by cervical dislocation, and tumors were excised.

### NSG AML xenografts.

Eight-week-old mice (The Jackson Laboratory, stock no. 005557) were injected via the tail vein with 2 million KG-1 cells. Engraftment was assessed at 2 weeks by tail vein bleeding and flow cytometry for huCD45 expression. Animals with >2% CD45 expression in peripheral blood mononuclear cells were included in the experiment. A total of 18 animals were randomized into 2 groups and treated with ixazomib 8 mg/kg or vehicle (5% cyclodextran) administered i.v. twice a week. Animals in both groups were treated for 4 weeks, after which PB, BM, and spleen were collected for analysis. Analysis of human cell engraftment in NSG mice by flow cytometry for huCD45 and histological and immunohistochemical analyses of the BM were performed. PB counts were assessed using the BRL Coulter Counter (Beckman Coulter). Protein and RNA were extracted from the BM of sacrificed animals for immunoblotting and mRNA analysis. Cytospin slides were also prepared from the BM MNCs at sacrifice, and FOXM1 IHC was performed as detailed above. The nuclear expression of FOXM1 was quantified by the Aperio scope and Halo software detailed above to compare treated and untreated groups.

### FLT3-ITD retroviral particle preparation.

Retrovirus supernatant was produced with the FLT3-ITD retroviral construct in the Platinum-E (Plat-E) retroviral packaging cell line by lipofectamin 2000 transfection according to standard protocol. The FLT3-ITD construct was provided by Philip Jost (Technical University of Munich, Munich, Germany).

### FLT3-ITD BM transplant model.

Rosa26-FoxM1b Tg, Arf ^–/–^ C57BL/6, and Arf^–/–^ C57BL/6 transgenic animals (25) were provided by Pradip Raychaudhuri (University of Illinois at Chicago) and bred in order to obtain the sufficient number of donor animals of each genotype. Eight-week-old donor mice were administered a single dose of 5’-fluorouracil (150 mg/kg, Fresenius Kabi) i.p. and subsequently harvested after 6 days by CO_2_ asphyxiation, followed by cervical dislocation. BM was flushed from femurs and tibias, and RBCs were lysed (Red Blood Cell Lysis, RBCL buffer, MilliporeSigma). Cells were cultured overnight with IL-3 (10 ng/ml, Peprotech), IL-6 (10 ng/ml, Peprotech), and stem cell factor (100 ng/ml, Peprotech) in StemSpan medium (Stemcell Technologies). Cells were transduced by 2 rounds of spin infection with retroviral supernatant carrying the FLT3-ITD-GFP oncogene. After this, 1 million cells, which included 200,000 support cells derived from a healthy donor, were washed in PBS, resuspended in HBSS, and injected (50 μl) retroorbitally into irradiated (11 Gy) recipient mice (C57BL/6J) 24 hours after irradiation.

Engraftment of the FLT3-ITD–transduced mouse BM cells was assessed at 2 weeks by analyzing PB obtained by tail nicking for GFP-positive leukocytes. Once disease was established (defined as >5% GFP expression in peripheral blood mononuclear cells), treatment began in all groups. Cytarabine (75 mg/kg, Hospira Worldwide Inc.) was injected i.p. for 5 consecutive days (days 1–5). Mice were monitored for 3 weeks following completion of treatment, and disease phenotype was compared between control- and cytarabine-treated groups with respect to genotype using bleeding to monitor blood counts and flow cytometry for leukemic burden. At the completion of the study, mice were sacrificed, and BM and spleen were harvested and analyzed for the presence of leukemia cells by flow cytometry. Spleen weights were obtained, and blood smears were prepared.

### Statistics.

For the clinical data on AML patients, OS time was measured from the date of diagnostic BM biopsy to death, with censorship at the date of last contact. Disease-free survival (DFS) was measured from the date of CR to the first relapse or death; patients alive and in CR were censored at the date of last contact. Two-tailed *t* tests (unless otherwise specified) or Kruskal-Wallis tests (for skewed variables) were used to compare means or medians of quantitative variables between response status, and χ^2^ test to compare categorical variables. Logistic regression was used to assess associations with CR. The Kaplan Meier method was used to describe OS, event-free survival (EFS), and DFS. Cox regression models were used to assess associations with these outcomes.

For the in vitro experiments, statistical analysis was performed using 1-way ANOVA followed by Tukey’s multiple comparison post test or unpaired 2-tailed *t* test. *P* < 0.05 was considered to be statistically significant.

### Study approval.

All methods involving patients were performed in accordance with the relevant guidelines and regulations set by the Northwestern and University of Illinois at Chicago IRBs. AML MNCs were obtained from patients with newly diagnosed or relapsed AML after informed consent using an IRB-approved protocol. All of the procedures that involved animals were in accordance with and approved by the Animal Care and Use Committee of the University of Illinois at Chicago. Experiments in NSG mice were performed at the Biologic Research Laboratory at the University of Illinois using an IACUC-approved protocol.

## Author contributions

IK and MH performed the experiments. AP, OF, RS, and NK collected clinical data on human subjects. YHC and NA reviewed patient BM slides prior to scanning. LL performed statistical analysis on clinical and imaging data. NM processed primary patient samples and provided expertise on leukemia xenograft models. JDC provided scientific expertise on translational leukemia models. IK, MH, and ALG analyzed the data. IK, MH, and ALG wrote the paper.

## Supplementary Material

Supplemental data

## Figures and Tables

**Table 2:**
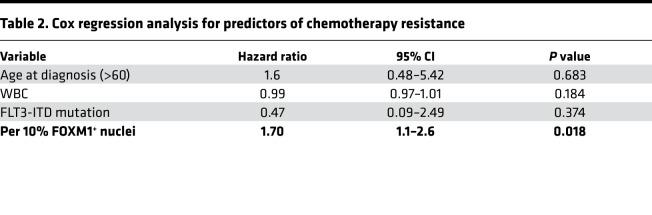
Cox regression analysis for predictors of chemotherapy resistance

**Table 1:**
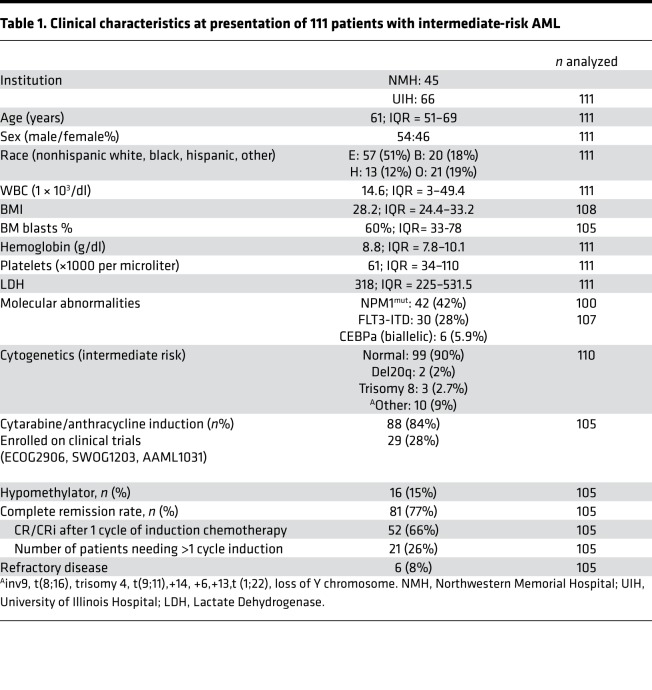
Clinical characteristics at presentation of 111 patients with intermediate-risk AML

## Version 1 08/09/2018

Electronic publication
