# Supplementary material for: FOXM1 contributes to treatment failure in acute myeloid leukemia
Source: JCI Insight. 2018 Aug 9;3(15):e121583. doi: 10.1172/jci.insight.121583 (PMC6129129; doi:10.1172/jci.insight.121583)

Supplemental Figure 1. **(A)** In a single institution survival analysis, high FOXM1 N:C ratio was associated with inferior overall survival as shown in the survival curves. **(B)** In a multivariate analysis incorporating FLT3-ITD, NPM1 mutation, BMI, WBC and age, FOXM1 N:C ratio had independent prognostic significance in predicting overall survival.

Supplemental Figure 2. FOXM1 diminishes response to anti-leukemic chemotherapy. (A-B) HL-60 and THP-1 cells were treated as indicated. Total cell lysates were analyzed by western blotting for the level of FOXM1. **(C)** Transgenic FOXM1 overexpressing (FoxM1b Tg;Arf−/−) and control (Arf−/−) animals were treated with 5-FU to enrich for hematopoietic progenitor cells. These cells were transduced with FLT3-ITD retroviral particles and transplanted into syngeneic recipients. Following disease establishment animals were randomized and treated with vehicle or cytarabine (AraC) for 5 consecutive days. Three weeks after treatment the peripheral blood chimerism was assessed by GFP measurement by flow cytometry. Data are expressed as the mean ± SEM (n=4/group); $P<0.05$ by unpaired two-tailed *t* test. **(D)** The spleens of AraC-treated and untreated syngeneic recipients generated as described in *C* were weighed. $P<0.05$ by unpaired two-tailed *t* test. **(E)** Blood smears following treatment show markedly increased circulating myeloid cells in the FOXM1 overexpressing animals (200X magnification). **(F)** Plot shows leukocyte burden does not diminish following treatment in the FOXM1 overexpressing mice. Data are expressed as the mean ± SEM (n=2-5/group); $P=0.058$ by unpaired one-tailed *t* test.

Supplemental Figure 3. Ixazomib inhibits FOXM1 activity. (A) Inhibition of colony formation was seen in MV-4-11 FOXM1 knockdown cells. Colonies were imaged with the EVOS XL Core Imaging System using the 4X objective. **(B)** Plot shows the mean +/- SEM of a representative

colony assay experiment plated in triplicate. *P*<0.05 by unpaired two-tailed *t* test. **(C)** Ixazomib treated and untreated SET-2 leukemia cells were collected for RNA extraction. Quantitative real time PCR was carried out with FOXM1, AurkB, Cdc25B and Plk1 primers. Graphs show quantification as percentage of mRNA expression levels in treated cells compared to control cells, mean ± SEM of three independent experiments. *P*<0.05 by one-way ANOVA followed by Tukey's multiple comparison post test. **(D)** In SET-2 cells, FOXM1 protein expression was suppressed by treatment with Ixazomib as detected by immunoblotting. This also correlated with caspase-3 cleavage.

Supplemental Figure 4. Ixazomib is well tolerated and does not affect normal hematopoiesis. **(A-C)** NSG mice were treated with 8mg/kg Ixazomib i.v. twice a week, 7 times total. Peripheral blood was analyzed to study the effects on normal blood production. Treated animals showed similar white blood cell, hemoglobin and platelet counts to control animals suggesting that Ixazomib does not affect normal hematopoiesis. Data are expressed as the mean ± SEM (n=4-5/group); *P*=ns by unpaired two-tailed *t* test. **(D)** Also, there was no difference in the weight of the treated and control animals during the treatment period.

<u>**Supplementary Figure 1**</u>

A)

FOXM1 Nuc:Cyt Ratio:

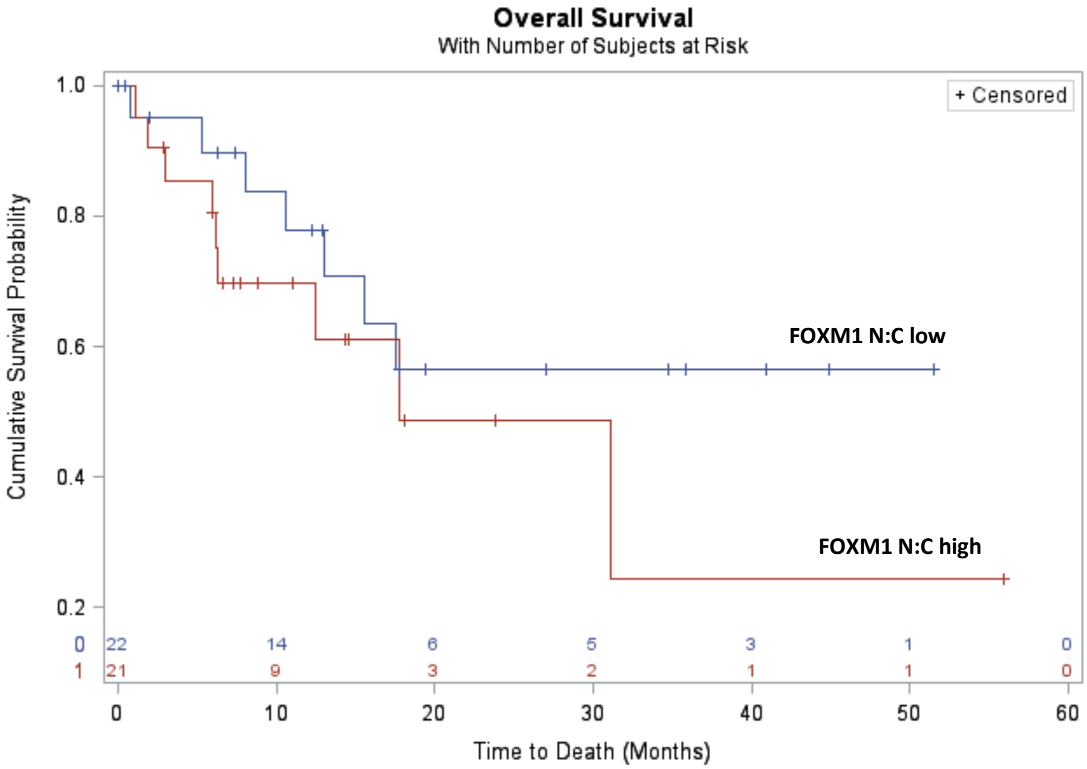

B)

Multi-variate analysis for OS (single institution):

Parameter	Pr > ChiSq	Hazard Ratio (95% CI)
**Age**	0.0154	6.821 (1.44, 32.25)
**WBC**	0.0141	1.012 (1.002, 1.022)
**BMI**	0.0010	1.404 (1.148, 1.718)
**FLT3-ITD mutation**	0.0934	3.196 (0.822, 12.418)
**NPM1 mutation**	0.1000	0.314 (0.079, 1.249)
**AvgFoxM1NucCytRatio per 0.1 Unit Increase**	0.0185	1.883 (1.112, 3.188)

Supplemental Fig. 2

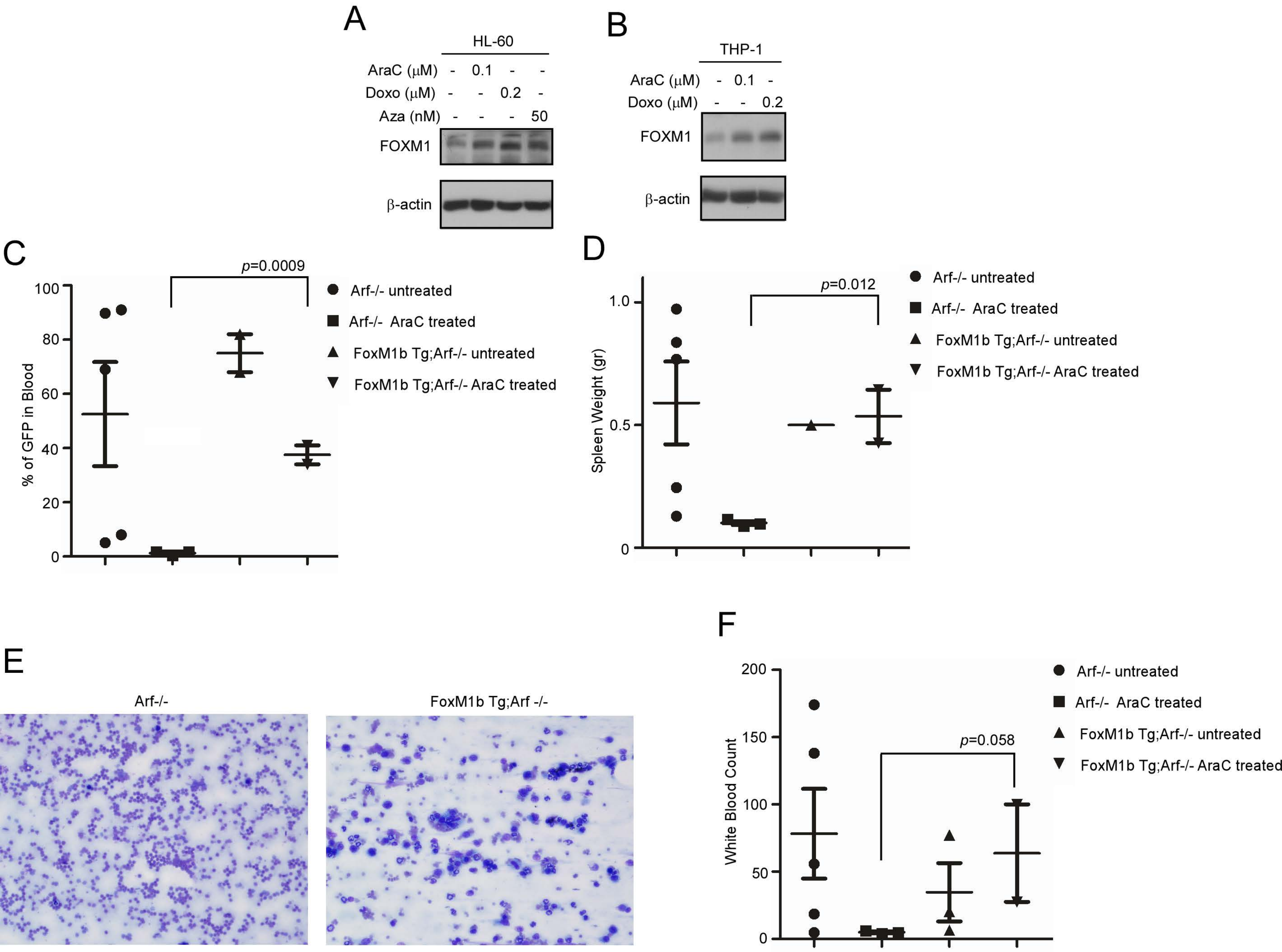

Supplemental Fig. 3

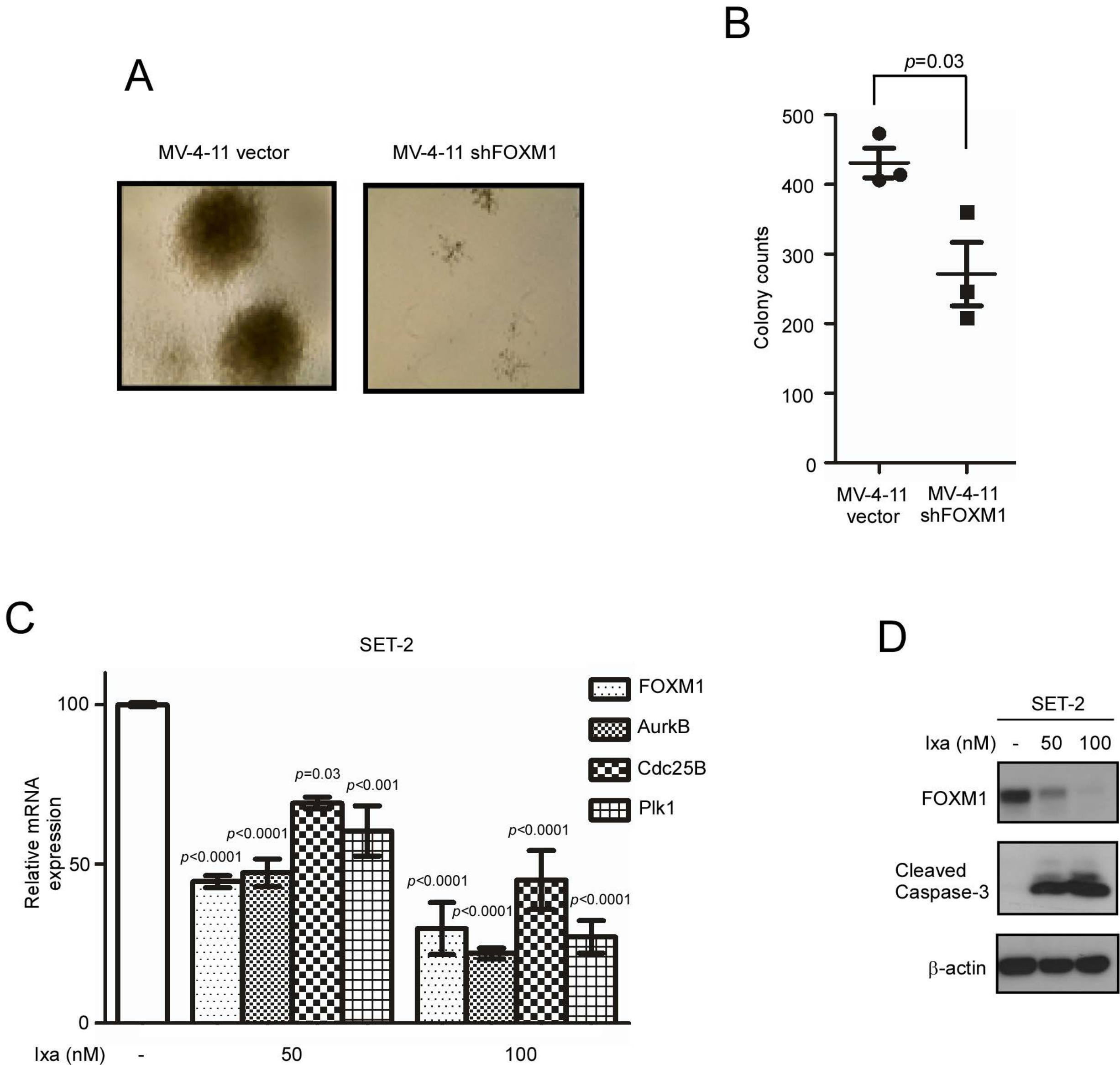

Supplemental Fig. 4

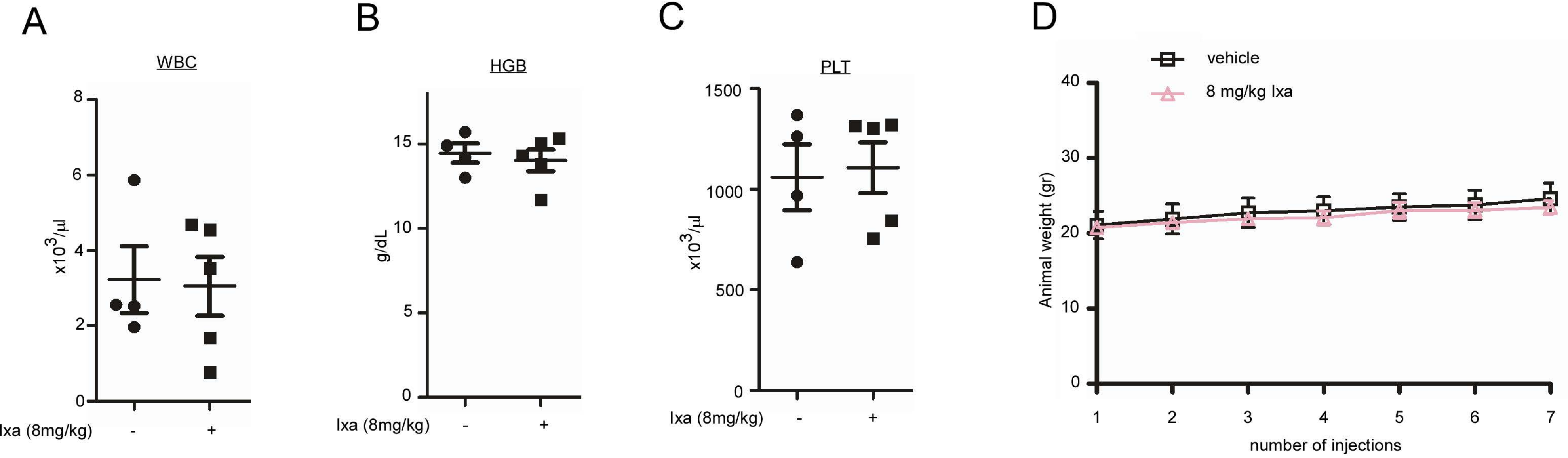

Supplement: Supplemental data [file jciinsight-3-121583-s190.pdf]
